# 2,2-Dimethyl-1,3-benzodioxol-4-yl *N*-methyl­carbamate

**DOI:** 10.1107/S1600536810036123

**Published:** 2010-09-18

**Authors:** Cheng-Cai Xia

**Affiliations:** a Department of Pharmaceutical Sciences, Taishan Medicine College, Taian 271000, People’s Republic of China

## Abstract

In the title compound, C_11_H_13_NO_4_, the two fused rings are almost coplanar, making a dihedral angle of 3.02 (8)°. In the crystal, chains are formed parallel to [010] through N—H⋯O hydrogen bonds between the amine and carbonyl groups.

## Related literature

For benzodioxole derivatives, see: Ullrich *et al.* (2004 ▶); Gates & Gillon (1974 ▶); Arndt & Franke (1977 ▶); Joshi *et al.* (2005 ▶); Jae *et al.* (2001 ▶); Leite *et al.* (2004 ▶).
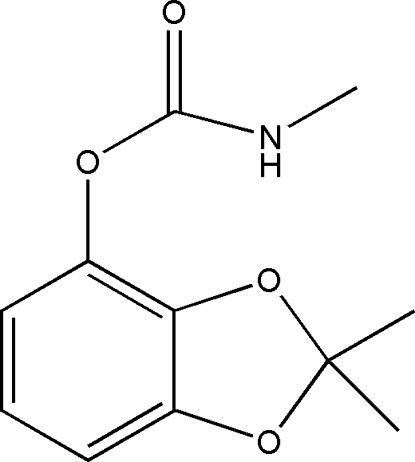


## Experimental

### 

#### Crystal data

C_11_H_13_NO_4_
*M* *_r_* = 223.22
Monoclinic, 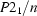

*a* = 9.505 (6) Å
*b* = 9.669 (7) Å
*c* = 12.355 (8) Å
β = 94.326 (11)°
*V* = 1132.2 (13) Å^3^
*Z* = 4
Mo *K*α radiation
μ = 0.10 mm^−1^
*T* = 298 K
0.24 × 0.18 × 0.16 mm

#### Data collection

Siemens SMART APEX CCD area-detector diffractometer
Absorption correction: multi-scan (*SADABS*; Siemens, 1996 ▶) *T*_min_ = 0.976, *T*_max_ = 0.984
5692 measured reflections
2003 independent reflections
1615 reflections with *I* > 2σ(*I*)
*R*_int_ = 0.023

#### Refinement

*R*[*F* ^2^ > 2σ(*F* ^2^)] = 0.035
*wR*(*F* ^2^) = 0.098
*S* = 1.03
2003 reflections
146 parameters
H-atom parameters constrained
Δρ_max_ = 0.19 e Å^−3^
Δρ_min_ = −0.13 e Å^−3^

### 

Data collection: *SMART* (Siemens, 1996 ▶); cell refinement: *SAINT* (Siemens, 1996 ▶); data reduction: *SAINT*; program(s) used to solve structure: *SHELXS97* (Sheldrick, 2008 ▶); program(s) used to refine structure: *SHELXL97* (Sheldrick, 2008 ▶); molecular graphics: *SHELXTL* (Sheldrick, 2008 ▶); software used to prepare material for publication: *SHELXTL*.

## Supplementary Material

Crystal structure: contains datablocks global, I. DOI: 10.1107/S1600536810036123/bh2301sup1.cif

Structure factors: contains datablocks I. DOI: 10.1107/S1600536810036123/bh2301Isup2.hkl

Additional supplementary materials:  crystallographic information; 3D view; checkCIF report

## Figures and Tables

**Table 1:** Hydrogen-bond geometry (Å, °)

*D*—H⋯*A*	*D*—H	H⋯*A*	*D*⋯*A*	*D*—H⋯*A*
N1—H1⋯O1^i^	0.86	2.01	2.819 (3)	157

Symmetry code: (i) 
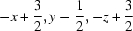
.

**Fig. 1.**
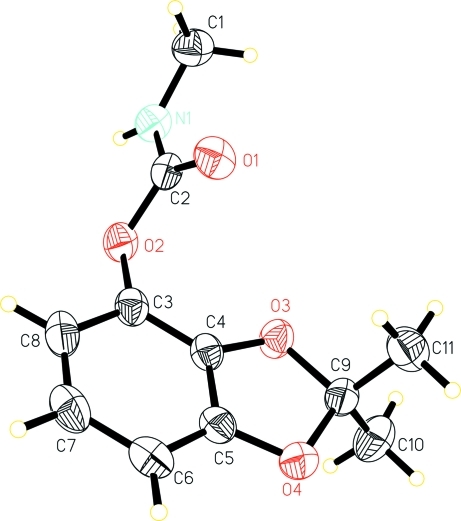
The asymmetric unit of the title molecule with atom labels, showing 40% probability displacement ellipsoids.

**Fig. 2.**
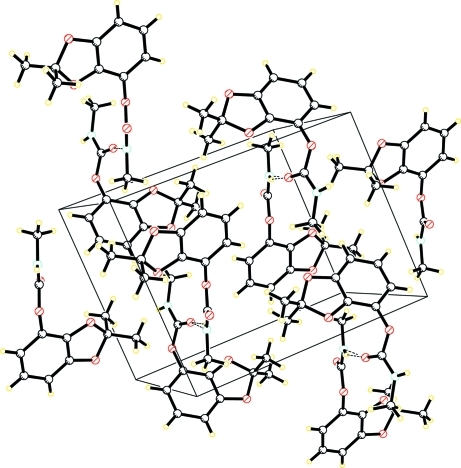
Part of the crystal structure, with hydrogen bonds shown as dashed lines.

## supplementary crystallographic information

### Comment

Benzodioxoles derivatives can be used as inhibitors of mono-oxygenase enzymes (Ullrich *et al.*, 2004), pesticides or pesticide intermediates (Gates & Gillon, 1974), herbicides (Arndt & Franke, 1977), antioxidants (Joshi *et al.*, 2005), antimicrobials (Jae *et al.*, 2001) and medicines (Leite *et al.*, 2004). As a part of our continuing interest in the synthesis of benzodioxole derivatives, we have isolated the title compound from the reaction of isocyanatomethane, triethylamine and 2,2-dimethylbenzo[*d*][1,3]dioxol-4-ol, as colorless crystals suitable for X-ray analysis.

As shown in Fig. 1, the molecule is built-up of a five-membered ring and a six-membered ring. Atoms C4, O3, O4, C5, and C6 are coplanar, which is illustrated clearly by the torsion angle O3—C4—C5—C6 [-179.39 (14)°], C3—C4—C5—O4 [177.83 (13)°], C3—C4—C5—C6 [-0.5 (2)°], and O3—C4—C5—O4 [-1.11 (17)°]. In the crystal structure, amino groups and carbonyl groups are involved in the hydrogen-bond network. Carbonyl atom O1 acts as an acceptor, forming the intramolecular hydrogen bonds depicted in Fig. 2.

### Experimental

The title compound was synthesized from a mixture of triethylamine (2 mmol, 0.2 g), isocyanatomethane (0.11 mol, 6.3 g) and 2,2-dimethylbenzo[*d*][1,3]dioxol-4-ol (0.1 mol, 16.6 g). The resulting compound was dissolved in 20 ml of ethanol and 2 ml of water, and refluxed for 10 min. The system was cooled to room temperature and colorless crystals were collected after two weeks.

### Refinement

Amine H atom H1 was found in a difference map, and its position fixed. Other H atoms were placed in calculated positions and allowed to ride on their parent atoms at distances of 0.93 (aromatic CH) or 0.96 Å (methyl CH_3_), with *U*_iso_(H) values fixed to 1.2 or 1.5 times *U*_eq_ of the parent atoms.

### Crystal data

**Table:**

C_11_H_13_NO_4_	*F*(000) = 472
*M**_r_* = 223.22	*D*_x_ = 1.310 Mg m^−^^3^
Monoclinic, *P*2_1_/*n*	Melting point: 408 K
Hall symbol: -P 2yn	Mo *K*α radiation, λ = 0.71073 Å
*a* = 9.505 (6) Å	Cell parameters from 2406 reflections
*b* = 9.669 (7) Å	θ = 2.7–26.7°
*c* = 12.355 (8) Å	µ = 0.10 mm^−^^1^
β = 94.326 (11)°	*T* = 298 K
*V* = 1132.2 (13) Å^3^	Block, colorless
*Z* = 4	0.24 × 0.18 × 0.16 mm

### Data collection

**Table:**

Siemens SMART APEX CCD area-detector diffractometer	2003 independent reflections
Radiation source: fine-focus sealed tube	1615 reflections with *I* > 2σ(*I*)
graphite	*R*_int_ = 0.023
φ and ω scans	θ_max_ = 25.1°, θ_min_ = 2.7°
Absorption correction: multi-scan (*SADABS*; Siemens, 1996)	*h* = −11→10
*T*_min_ = 0.976, *T*_max_ = 0.984	*k* = −11→11
5692 measured reflections	*l* = −13→14

### Refinement

**Table:**

Refinement on *F*^2^	Secondary atom site location: difference Fourier map
Least-squares matrix: full	Hydrogen site location: inferred from neighbouring sites
*R*[*F*^2^ > 2σ(*F*^2^)] = 0.035	H-atom parameters constrained
*wR*(*F*^2^) = 0.098	*w* = 1/[σ^2^(*F*_o_^2^) + (0.0465*P*)^2^ + 0.1766*P*] where *P* = (*F*_o_^2^ + 2*F*_c_^2^)/3
*S* = 1.03	(Δ/σ)_max_ < 0.001
2003 reflections	Δρ_max_ = 0.19 e Å^−^^3^
146 parameters	Δρ_min_ = −0.13 e Å^−^^3^
0 restraints	Extinction correction: *SHELXL97* (Sheldrick, 2008), Fc^*^=kFc[1+0.001xFc^2^λ^3^/sin(2θ)]^-1/4^
0 constraints	Extinction coefficient: 0.035 (3)
Primary atom site location: structure-invariant direct methods	

### Fractional atomic coordinates and isotropic or equivalent isotropic displacement parameters (Å^2^)

**Table:**

	*x*	*y*	*z*	*U*_iso_*/*U*_eq_	
O1	0.78908 (12)	0.22049 (10)	0.73401 (10)	0.0666 (3)	
O2	0.91170 (11)	0.04289 (10)	0.81309 (9)	0.0614 (3)	
O3	1.07164 (11)	0.14292 (11)	0.63309 (8)	0.0624 (3)	
O4	1.27901 (11)	0.26676 (13)	0.65853 (9)	0.0707 (4)	
N1	0.69122 (14)	0.01063 (12)	0.74902 (10)	0.0589 (4)	
H1	0.7052	−0.0723	0.7726	0.071*	
C1	0.55627 (18)	0.04608 (19)	0.69670 (16)	0.0748 (5)	
H1A	0.5096	0.1103	0.7412	0.112*	
H1B	0.4999	−0.0360	0.6864	0.112*	
H1C	0.5689	0.0876	0.6275	0.112*	
C2	0.79399 (16)	0.10118 (14)	0.76185 (11)	0.0491 (4)	
C3	1.03134 (16)	0.12582 (15)	0.82661 (12)	0.0537 (4)	
C4	1.10295 (15)	0.16741 (14)	0.74063 (11)	0.0505 (4)	
C5	1.22535 (16)	0.24235 (16)	0.75644 (12)	0.0554 (4)	
C6	1.28072 (19)	0.27957 (18)	0.85695 (14)	0.0674 (5)	
H6	1.3632	0.3313	0.8667	0.081*	
C7	1.2084 (2)	0.23669 (19)	0.94372 (14)	0.0730 (5)	
H7	1.2435	0.2597	1.0137	0.088*	
C8	1.0863 (2)	0.16103 (17)	0.92966 (13)	0.0670 (5)	
H8	1.0402	0.1333	0.9898	0.080*	
C9	1.17105 (17)	0.22577 (17)	0.57665 (13)	0.0602 (4)	
C10	1.2336 (2)	0.1358 (2)	0.49544 (16)	0.0884 (6)	
H10A	1.3010	0.1879	0.4581	0.133*	
H10B	1.1604	0.1029	0.4441	0.133*	
H10C	1.2798	0.0586	0.5316	0.133*	
C11	1.0989 (2)	0.35311 (19)	0.53297 (16)	0.0795 (5)	
H11A	1.0580	0.4014	0.5908	0.119*	
H11B	1.0260	0.3280	0.4785	0.119*	
H11C	1.1662	0.4119	0.5015	0.119*	

### Atomic displacement parameters (Å^2^)

**Table:**

	*U*^11^	*U*^22^	*U*^33^	*U*^12^	*U*^13^	*U*^23^
O1	0.0786 (8)	0.0347 (6)	0.0860 (8)	0.0026 (5)	0.0022 (6)	0.0060 (5)
O2	0.0715 (7)	0.0454 (6)	0.0679 (7)	0.0011 (5)	0.0085 (5)	0.0166 (5)
O3	0.0668 (7)	0.0740 (7)	0.0466 (6)	−0.0115 (5)	0.0049 (5)	−0.0029 (5)
O4	0.0564 (6)	0.0921 (9)	0.0635 (7)	−0.0089 (6)	0.0032 (5)	0.0079 (6)
N1	0.0698 (8)	0.0357 (6)	0.0725 (9)	−0.0011 (6)	0.0139 (7)	0.0037 (6)
C1	0.0679 (11)	0.0654 (11)	0.0919 (13)	−0.0016 (9)	0.0109 (10)	0.0018 (9)
C2	0.0669 (9)	0.0353 (7)	0.0470 (8)	0.0064 (7)	0.0154 (7)	−0.0002 (6)
C3	0.0642 (9)	0.0450 (8)	0.0520 (9)	0.0082 (7)	0.0046 (7)	0.0053 (6)
C4	0.0594 (9)	0.0461 (8)	0.0454 (8)	0.0054 (7)	0.0009 (7)	−0.0001 (6)
C5	0.0547 (9)	0.0559 (8)	0.0547 (9)	0.0069 (7)	−0.0012 (7)	0.0018 (7)
C6	0.0635 (10)	0.0667 (11)	0.0695 (11)	0.0067 (8)	−0.0119 (9)	−0.0060 (8)
C7	0.0874 (13)	0.0752 (11)	0.0536 (10)	0.0159 (10)	−0.0133 (9)	−0.0085 (8)
C8	0.0877 (12)	0.0655 (10)	0.0479 (9)	0.0156 (9)	0.0055 (8)	0.0062 (7)
C9	0.0618 (9)	0.0676 (10)	0.0516 (9)	−0.0041 (8)	0.0069 (7)	0.0054 (7)
C10	0.1012 (15)	0.0889 (14)	0.0794 (13)	−0.0004 (11)	0.0356 (11)	−0.0040 (10)
C11	0.0805 (12)	0.0732 (12)	0.0827 (13)	0.0011 (10)	−0.0081 (10)	0.0106 (9)

### Geometric parameters (Å, °)

**Table:**

O1—C2	1.2037 (18)	C4—C5	1.372 (2)
O2—C2	1.3650 (18)	C5—C6	1.360 (2)
O2—C3	1.3911 (19)	C6—C7	1.380 (3)
O3—C4	1.3603 (19)	C6—H6	0.9300
O3—C9	1.4560 (19)	C7—C8	1.372 (3)
O4—C5	1.368 (2)	C7—H7	0.9300
O4—C9	1.441 (2)	C8—H8	0.9300
N1—C2	1.312 (2)	C9—C10	1.485 (2)
N1—C1	1.434 (2)	C9—C11	1.490 (2)
N1—H1	0.8600	C10—H10A	0.9600
C1—H1A	0.9600	C10—H10B	0.9600
C1—H1B	0.9600	C10—H10C	0.9600
C1—H1C	0.9600	C11—H11A	0.9600
C3—C4	1.365 (2)	C11—H11B	0.9600
C3—C8	1.382 (2)	C11—H11C	0.9600
			
C2—O2—C3	116.85 (11)	C7—C6—H6	121.7
C4—O3—C9	105.66 (12)	C8—C7—C6	121.86 (16)
C5—O4—C9	106.26 (12)	C8—C7—H7	119.1
C2—N1—C1	121.84 (14)	C6—C7—H7	119.1
C2—N1—H1	119.1	C7—C8—C3	120.35 (16)
C1—N1—H1	119.1	C7—C8—H8	119.8
N1—C1—H1A	109.5	C3—C8—H8	119.8
N1—C1—H1B	109.5	O4—C9—O3	105.58 (12)
H1A—C1—H1B	109.5	O4—C9—C10	109.58 (15)
N1—C1—H1C	109.5	O3—C9—C10	107.98 (14)
H1A—C1—H1C	109.5	O4—C9—C11	108.10 (14)
H1B—C1—H1C	109.5	O3—C9—C11	109.30 (14)
O1—C2—N1	126.32 (15)	C10—C9—C11	115.83 (16)
O1—C2—O2	122.75 (14)	C9—C10—H10A	109.5
N1—C2—O2	110.93 (13)	C9—C10—H10B	109.5
C4—C3—C8	117.95 (16)	H10A—C10—H10B	109.5
C4—C3—O2	121.84 (13)	C9—C10—H10C	109.5
C8—C3—O2	120.08 (14)	H10A—C10—H10C	109.5
O3—C4—C3	128.60 (14)	H10B—C10—H10C	109.5
O3—C4—C5	110.59 (13)	C9—C11—H11A	109.5
C3—C4—C5	120.80 (14)	C9—C11—H11B	109.5
C6—C5—O4	128.11 (16)	H11A—C11—H11B	109.5
C6—C5—C4	122.36 (15)	C9—C11—H11C	109.5
O4—C5—C4	109.50 (13)	H11A—C11—H11C	109.5
C5—C6—C7	116.66 (17)	H11B—C11—H11C	109.5
C5—C6—H6	121.7		
			
C1—N1—C2—O1	0.8 (2)	C3—C4—C5—C6	−0.5 (2)
C1—N1—C2—O2	−179.66 (13)	O3—C4—C5—O4	−1.11 (17)
C3—O2—C2—O1	−3.81 (19)	C3—C4—C5—O4	177.83 (13)
C3—O2—C2—N1	176.62 (12)	O4—C5—C6—C7	−177.16 (15)
C2—O2—C3—C4	−68.19 (17)	C4—C5—C6—C7	0.8 (2)
C2—O2—C3—C8	116.03 (15)	C5—C6—C7—C8	−0.4 (3)
C9—O3—C4—C3	172.56 (15)	C6—C7—C8—C3	−0.3 (3)
C9—O3—C4—C5	−8.60 (16)	C4—C3—C8—C7	0.6 (2)
C8—C3—C4—O3	178.46 (14)	O2—C3—C8—C7	176.56 (14)
O2—C3—C4—O3	2.6 (2)	C5—O4—C9—O3	−15.26 (16)
C8—C3—C4—C5	−0.3 (2)	C5—O4—C9—C10	−131.32 (15)
O2—C3—C4—C5	−176.13 (13)	C5—O4—C9—C11	101.61 (15)
C9—O4—C5—C6	−171.39 (16)	C4—O3—C9—O4	14.55 (15)
C9—O4—C5—C4	10.45 (17)	C4—O3—C9—C10	131.69 (14)
O3—C4—C5—C6	−179.39 (14)	C4—O3—C9—C11	−101.52 (15)

### Hydrogen-bond geometry (Å, °)

**Table:**

*D*—H···*A*	*D*—H	H···*A*	*D*···*A*	*D*—H···*A*
N1—H1···O1^i^	0.86	2.01	2.819 (3)	157

Symmetry codes: (i) −*x*+3/2, *y*−1/2, −*z*+3/2.
